# Temporal choroidal fissure cyst: a rare cause of temporal lobe epilepsy

**DOI:** 10.11604/pamj.2020.36.120.21327

**Published:** 2020-06-23

**Authors:** Asma Achour, Walid Mnari, Ahmed Miladi, Badii Hmida, Mezri Maatouk, Mondher Golli, Ahmed Zrig

**Affiliations:** 1 Radiology department A, Fattouma Bourguiba Hospital, Monastir Medical University, Monastir, Tunisia

**Keywords:** Choroidal fissure cyst, seizures, epilepsy

## Abstract

Only a few cases of temporal choroidal fissure cyst are reported. We describe a new case of an 8 years old girl who manifested complex partial seizure. The diagnosis was made by magnetic resonance imagining (MRI). The signal intensity of the cyst was identical to the cerebrospinal fluid (CSF), and the underlying hippocampus was compressed by the cyst. The seizures were medically controlled. The value of MRI in the diagnosis and medical treatment will be discussed.

## Introduction

Choroidal fissure cysts are rare and often incidentally discovered [1]. There are a few cases reported and they are usually asymptomatic. The relationship between complex partial seizure and temporal fissure cyst is controversial [2]. We describe a new case of a partial complex seizure that might be explained by a right temporal choroidal fissure cyst. We emphasize the value of MRI in the diagnosis and to differentiate these findings from intra axial cystic lesions of the temporal lobe.

## Patient and observation

**Figure 1:**
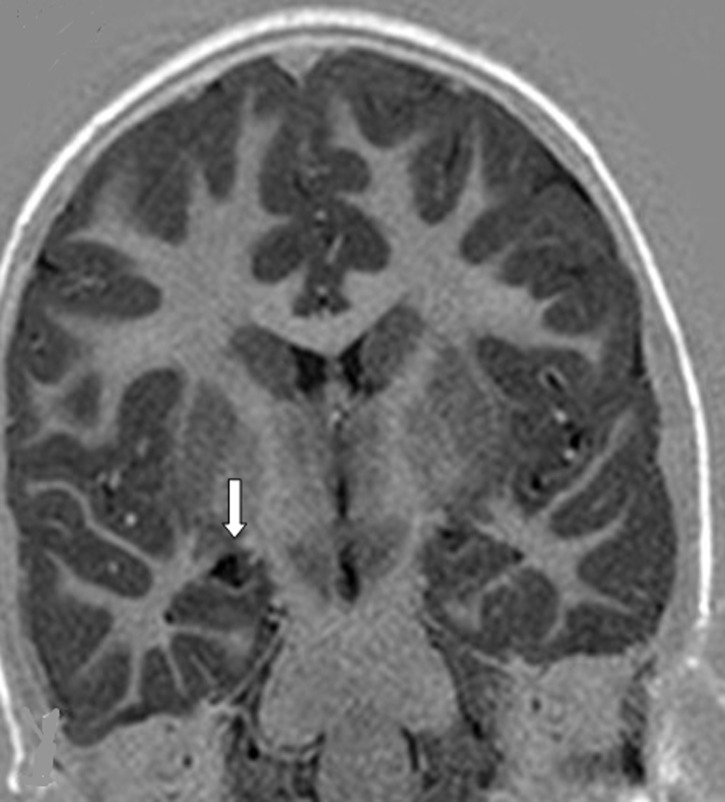
coronal MRI T1 inversion recovery showing a round cystic lesion of the right choroidal fissure compressing the hippocampus, hyposignal T1 (arrow)

**Figure 2:**
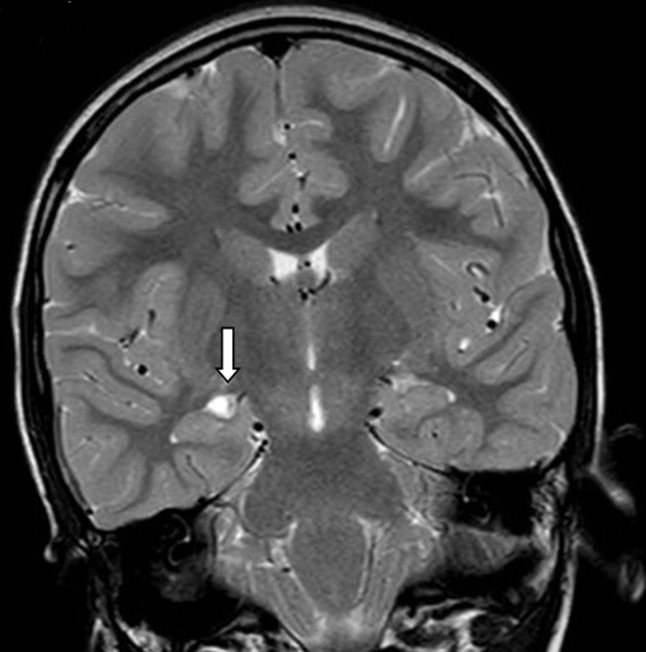
coronal MRI T2: same lesion, hypersignal T2 (arrow)

**Figure 3:**
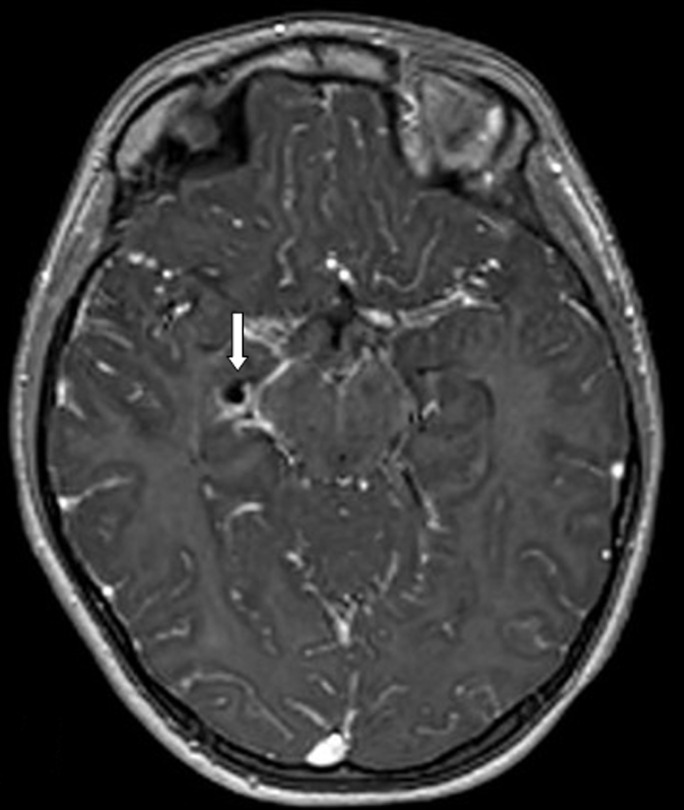
axial MRI T1 after injection of gadolinium: cystic lesion without peripheral enhancement (arrow)

Our patient was an 8 years old child with a history of epilepsy. She experienced her first seizure at the age of 5 years. She had a normal neuropsychological development. There was no family history of epilepsy. The physical examination was normal. Her seizures began with what it was described as «weird feeling» and then followed by automatism (chewing). This seizure lasted about 1 minute and 30 seconds. Our patient expressed total amnesia of her seizures. 1.5 Tesla MRI revealed a small round cyst in the right choroidal fissure of the temporal horn compressing the hippocampus. The signal intensity of the cyst was hyposignal T1 (Figure 1), hypersignal T2 (Figure 2), no restriction of apparent diffusion coefficient (ADC), identical to that of the cerebrospinal fluid (CSF). There was no peripheral contrast enhancement or surrounding edema (Figure 3). Based on the MR features, the presumptive diagnosis of a choroidal fissure cyst was made and seizures were medically controlled by Carbamazepine.

## Discussion

The choroidal fissure is a cleft in the medial part of the lateral ventricle between the fornix and the thalamus [3]. Developmental errors may occur at the time of formation of primitive choroid plexus anywhere along the choroid fissure, thus forming a cyst. Small asymptomatic choroid plexus cysts are frequent incidental findings at autopsy [2]. There have been several reports concerning the relationship between the choroid plexus cyst and the epileptic syndrome. In Sherman *et al*. study, 5 patients of 26 who had cysts in or near the choroidal fissure had epilepsy but they concluded to incidental findings unrelated to patient symptomatology since they have no clinical correlation. Isolan *et al*. however, described two patients with choroidal fissure cysts and complex partial seizures ipsilateral to the cysts [4].

Morioka *et al*. describes also two other cases of choroidal fissure cysts associated with seizures [4]. In all the cases of choroidal fissure cysts, complex partial seizures were controlled medically as in our case. The study of the features of the content of these cystic intracranial lesions by means of traditional MR techniques (T1-weighted, T2-weighted, FLAIR and diffusion, are the key to distinct between intracranial cysts with a free water-like content versus those filled with a non-free water-like substance [5]. Choroidal fissure cysts are often small, homogeneous, signal identical to CSF in all the sequences [6]. On the other hand, non-free water-like substance such as neoplastic or cystic neoplasm, dermoid cysts, epidermoid cysts, abscess, and parasitic cysts will have a different signal from CSF because of the accumulation either of necrotic or of intercellular myxoid or proteinaceous material [5, 7].

## Conclusion

This article is written on the base of the occurrence of seizures in an 8-year-old child who led to the discovery of this usually benign cerebral lesion, which is known asymptomatic. Very rarely as for our patient the choroidal fissure cyst may cause seizures due to mass effect on the temporal lobe. We believe that further series should be made to prove the relationship between these types of cysts and complex partial seizure.
